# Sex-specific cardiac cardiolipin remodelling after doxorubicin treatment

**DOI:** 10.1186/s13293-015-0039-5

**Published:** 2015-10-15

**Authors:** Maryline Moulin, Audrey Solgadi, Vladimir Veksler, Anne Garnier, Renée Ventura-Clapier, Pierre Chaminade

**Affiliations:** UMR-S 1180, Inserm, Univ Paris-Sud, Université Paris-Saclay, Châtenay-Malabry, France; SFR IPSIT (Institut Paris-Saclay d’Innovation Thérapeutique), Univ Paris-Sud, Université Paris-Saclay, Châtenay-Malabry, France; UMS IPSIT Service d’Analyse des Médicaments et Métabolites, Châtenay-Malabry, France; Lip(Sys)2 ex EA4041, Univ Paris-Sud, Université Paris-Saclay, Châtenay-Malabry, France; Current address: Université Paris Diderot, Unité de Biologie Fonctionnelle et Adaptative, CNRS UMR 8251, Paris, France

**Keywords:** Cardiolipin, Sex-specific, Heart failure, Doxorubicin, LC/MS^n^

## Abstract

**Background:**

Imbalance in lipid metabolism and membrane lipid homeostasis has been observed in numerous diseases including heart failure and cardiotoxicity. Growing evidence links phospholipid alterations especially cardiolipins (CLs) to defects in mitochondrial function and energy metabolism in heart failure. We have shown recently that doxorubicin cardiotoxicity is more severe in male than female Wistar rats. We aimed to study whether this sex specificity is linked to differences in cardiac phospholipid profiles.

**Results:**

Adult male and female rats were injected 2 mg/kg doxorubicin weekly for 7 weeks. Cardiac phospholipid molecular species were determined by liquid chromatography coupled with mass spectrometry fragmentation (LC)/MS^n^. Sex difference in phosphatidylethanolamine and phosphatidylcholine species containing docosahexaenoic and docosapentaenoic acyl chains was observed, females having more than males. In both sexes, doxorubicin induced an important loss of the main CL(18:2)_4_, while the level of monolysocardiolipin MLCL(18:2)_3_ remained stable. However, a severe remodelling appeared in treated rats with the longest CL acyl chains in doxorubicin-treated females, which might compensate for the loss of tetra-linoleoyl CL. The level of oxidized cardiolipin was not particularly increased after doxorubicin treatment. Finally, expression of genes involved in the biosynthesis of fatty acid appeared to be decreased in doxorubicin-treated males.

**Conclusions:**

These results emphasize for the first time the cardiac remodelling in the phospholipid classes after doxorubicin treatment. These observations suggest that doxorubicin has a sex-specific impact on the heart phospholipidome especially on cardiolipin, an essential mitochondrial lipid. Further studies are needed to better understand the roles of lipids in the anthracycline cardiotoxicity and sex differences, but phospholipid cardioprotection seems a valuable new additive therapeutic strategy for anthracycline cardiotoxicity.

**Electronic supplementary material:**

The online version of this article (doi:10.1186/s13293-015-0039-5) contains supplementary material, which is available to authorized users.

## Background

Doxorubicin (DOXO), a widespread anti-cancer agent, has clinical limited use due to important deleterious cardiac side effects that ultimately lead to heart failure (HF). How anthracyclines induce both anti-cancer activity and cardiotoxicity are not completely understood [1]. Numerous studies have shown the interaction of doxorubicin with membranes, particularly cardiolipin (CL), causing membrane fluidity alteration and enzyme activity deficiency [2–4]. We have recently observed that after 7 weeks of doxorubicin treatment, male rats but not females had high mortality rate, signs of heart failure, and altered mitochondrial function, and we identified a significant decrease in total cardiolipin content specifically in the heart of treated males [5].

Phospholipids (PLs), a fundamental class of biological compounds, are the main component of membranes which represents a biological and physical barrier but also a site for interaction and signal transduction. Fatty acid (FA) composition of PL influences membrane characteristics such as membrane fluidity, microdomain formation, transport system, and membrane-bound enzyme activities (for review, see [6]). Obviously, deregulation of PL is involved in numerous diseases such as cancer, neurological disorders, hepatic disorders, and cardiovascular diseases (reviewed in [7, 8]).

Cardiolipin, a signature PL of the inner membrane of the mitochondria (around 20 % of the lipid composition), is involved in numerous functional and structural features of membrane-bound mitochondrial proteins and is linked to important mitochondrial processes, including apoptosis, mitochondrial dynamics, contact sites formation and assembly, and function of mitochondrial membrane proteins for an effective oxidative phosphorylation (reviewed in [9–12]). CL is a unique dimeric phospholipid containing two phosphatidyl moieties bridged by a glycerol and four fatty acyl chains. The FA composition is also highly specific and is predominantly composed of 18:2 acyl chains with tetra-linoleoyl cardiolipin (L_4_CL) being the most abundant [13]. CL composition depends on de novo synthesis of nascent CL followed by complex processes of acyl chain remodelling. The fatty acyl chain composition of CL is critical for mitochondrial function and cardiac pathology. Alteration in CL content and composition has been associated with numerous disorders like diabetes, thyroid status, aging, and heart failure [14].

HF is characterized by the inability of the heart to fulfill the metabolic requirements of the organism. Among the characteristics of HF, the state of energy depletion [15, 16], alterations of the mitochondrial function, and biogenesis [17] have been well described, but changes in cardiac PL composition are less addressed. However, cardiolipin may play a role in mitochondrial alterations in heart failure [10]. Indeed, decreased cardiolipin content, altered cardiolipin synthesis and remodelling [18], reduction in 18:2 CL species [10, 18, 19], and modified expression or activity of cardiac biosynthesis enzymes [20] have been reported in numerous diseases. The importance of CL in heart failure is exemplified by the Barth syndrome, a severe X-linked genetic disorder which is characterized by CL alterations, mitochondrial defects, and heart failure and which results from mutations in the *Tafazzin* gene, an acyltransferase involved in CL remodelling [21]. Finally, CLs are susceptible to oxidative damages because they are present in the mitochondria which are the main source of reactive oxygen species (ROS) and because they contain double bonds that are susceptible to oxidation. CL oxidation has been shown to induce decreased activity and organisation of respiratory chain complexes and to favor apoptosis (for review, see [11]).

As we have observed, a decrease in total cardiolipin content in treated males but no females; the objective of the present study was thus to explore in more detail the cardiac phospholipidome after doxorubicin treatment in adult rats and to understand whether CL remodelling participates in the sex-specific cardiac alterations.

## Methods

An expanded method section is available in the Additional file 1.

### Doxorubicin animal model

A chronic model of doxorubicin [22] by weekly intravenous injection of 2 mg/kg doxorubicin or saline solution during 7 weeks in 11-week-old male and female Wistar rats (Janvier Labs) was chosen as previously described [23]. Animal experimental procedures were approved by the Animal Ethics Committee of Paris Sud University. Investigations were done in accordance with European Community legislation relating to the care and use of animals (Directive 2010/63/EU) and the corresponding French legislation (French decree 2013-118 du 1er février 2013).

### Lipid extraction

A Folch method [24] was used to extract the lipids from 10 to 20 mg of heart homogenized in phosphate-buffered saline (PBS). Each group was composed of four animals; one technical repeat was done for a non-treated male and a doxorubicin-treated male. Total lipids were extracted by adding 1.5 mL of methanol and 3 mL of chloroform to the tissue suspension. After centrifugation at 1000*g* for 10 min, the lower phase containing total lipids was collected and evaporated to dryness at room temperature under nitrogen gas. The samples were resuspended in 100 μL of chloroform per 10 mg of heart and subsequently analyzed.

### Cardiolipin oxidation

Cardiolipins were oxidized following the method of Paradies [25]. Briefly, cardiolipins in PBS were mixed in 1 mL of oxygenated buffer containing 20 μM ferrous sulfate, 120 μM ADP, and 200 μM ascorbic acid for 15 min at 37 °C before being extracted by a Folch method.

### Phospholipid analysis

For the phospholipid analysis, each sample was duplicated, except the technical repeat for a non-treated male. Chromatographic system from Thermo Fisher Scientific included a Dionex U 3000 RSLC system with two quaternary pumps, an autosampler, and a column oven. The RSLC system was coupled online to a charged aerosol detector Corona CAD Ultra for the quantitative part of the study and a LTQ Orbitrap Velos Pro for the lipidomic study (all from Thermo Fisher).

#### Liquid chromatography

The separation of lipids was carried out on a PVA-Sil column (150 × 2.1 mm I.D., 120 A) (YMC Europe GmbH) with a 10 × 2 mm guard column packed with the same material both supplied by Interchim. The column temperature was thermostatically controlled at 35 °C. Chromatographic method was inspired from the method developed by Imbert et al. [26]. The flow rate was set at 400 μL/min, and a sample volume of 5 μL was injected. The mobile phase compositions were identical. An isopropanol phase (D) has been added to rinse the RSLC system. The solvent program has been modified as follows: the Corona CAD nebulizer was set at a temperature of 30 °C and the nitrogen pressure was set at 5 bar (Table 1).

**Table 1:** Chromatographic program with the following mobile phase compositions: mixture of heptane and isopropanol 98:2 (v/v) (phase A); mixture of chloroforme and isopropanol 65:35 (v/v) (phase B); mixture of methanol and water 95:5 (v/v) (phase C); isopropanol (phase D)

Time	*A*	*B*	*C*	*D*
*0*	98	2	0	0
*2*	98	2	0	0
*8*	12	88	0	0
*22*	0	60	40	0
*26*	0	60	40	0
*27*	0	0	0	100
*29*	0	0	0	100
*30*	98	2	0	0
*44*	98	2	0	0

All A, B and C mobile phases also contain acid acetic 1% (v/v) and triethylamine 0.08% (v/v)

The LC effluent was split after the analytical column with aid of a tee; thus, 250 μL/min entered to the Corona CAD and 150 μL/min entered the mass spectrometer. To increase the flow entering the Corona CAD and maintain good aerosol stability, absolute ethanol was added with a second mixing tee with a flow of 200 μL/min.

#### Mass spectrometry

The LTQ Orbitrap Velos Pro is equipped with an H-ESI II probe. Spray voltage was set at 3.3 kV. Heater temperature of the probe was set at 200 °C. Sheath gas, auxiliary gas, and sweep gas flow rates were set at 20, 8, and 0 (arbitrary unit), respectively. Capillary temperature was set at 325 °C and S-lens RF level at 60 %. Analysis was performed in negative mode to obtain structural nformation on phospholipids fatty chains. This mass spectrometer is equipped with two analyzers: a double linear ion trap (LTQ) for fragmentation at low resolution and an orbital trap for high-resolution detection. The detection was performed either in full mass spectrometry (MS) scan with 100,000 resolution and data dependant MS^2^ and MS^3^ with collision-induced dissociation in the CID fragmentation (collision energy set at 35).

#### Phospholipid species identification

The chromatographic method used separates the phospholipids by polar head. Retention time is a crucial information for polar head identification and compared to commercial standards. Then, species can be observed under each chromatographic peak. PL identification was performed as described [27] using high-resolution mass detection in full scan mode and MS^2^/MS^3^ fragmentation in data dependant mode. MS^n^ fragmentation has not always been performed by the instrument due to very small amount of some ions. When no MS^n^ data is available, PLs are mentioned as, e.g., PL(34:1). Otherwise, PL are mentioned as, e.g., PL(16:0-18:1). Sn1 and sn2 position of the acyl chains were not determined. Oxidized cardiolipin identification was performed as described by Kim et al. [28, 29].

#### Phospholipid species representation

Total phospholipid amount was determined with Corona CAD detection by calculating the concentration of each PL class (in micrograms per milliliter) compared to calibration ranges of commercial standards. PL species intensities were determined by taking the mean relative intensity of *m*/*z* ions over the chromatographic peak of each PL class.

#### 2D separation of CL

The sample’s cardiolipin was collected from 11 to 12 min, dried over nitrogen, and dissolved in 2D phase mobile (2-propanol:water:triethylamine:acetic acid 45:5:0.25:0.25). A Dionex C18 column (Acclaim RSLC 120, 2.2 μm, 120 Å, 2.1 × 150 mm) was used under isocratic solvent condition. The column temperature was thermostatically controlled at 40 °C. Flow was maintained at 200 μL/min on a Dionex U 3000 RSLC system, and MS and MS/MS acquisitions via LTQ Orbitrap Velos Pro were performed on the most abundant mass ions.

### Statistical analysis

MS raw data were submitted to chromatogram builder, alignment and gap filling using the MzMine 2.10 LC/MS toolbox [30]. SIMCA-P 12.0 was used to analyze the data generated by mass spectrometry (principal component analysis (PCA) and OPLS-DA). Data are expressed as mean ± SEM. Two-way ANOVA (homogenous variance verified by Levene test for all parameters except *Cds1* and *Acsl1*) was used to study the global effect of sex and treatment, followed by Student-Newman-Keuls post hoc test to determine individual differences. Statistical significance for doxorubicin effect was defined as * for *p* < 0.05, ** for *p* < 0.01, and *** for *p* < 0.001 and for sex effect as § for *p* < 0.05, §§ for *p* < 0.01, and §§§ for *p* < 0.001.

## Results

### Doxorubicin treatment affects cardiac phospholipid content differently between males and females

**Fig. 1:**
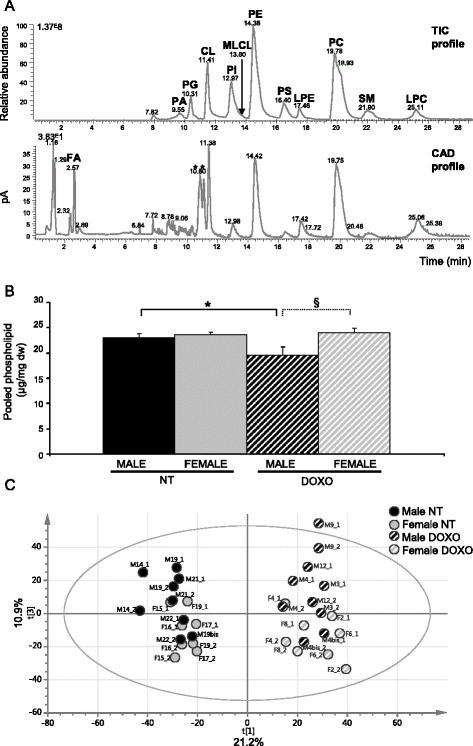
Doxorubicin and sex effect on cardiac phospholipids. **a** A representative phospholipid class separation (first dimension) obtained by LC-ESI/MS (*TIC*, total ion chromatogram) and Corona CAD detection; *asterisk* is for unspecific peak. **b** Phospholipid (CL/PI/PE/PS and PC) contents measured from the HPLC Corona CAD detection in ventricles of 7 weeks doxorubicin-treated rats. **c** Score plot obtained by principal component analysis (*PCA*) of *m*/*z* ions from cardiac tissue analysis generated by LC-ESI/MS. Each sample (four per group) was duplicated

Doxorubicin treatment induces a life-threatening cardiotoxicity with altered mitochondrial function. We have shown recently that males were less protected against DOXO cardiotoxicity and that cardiolipin content was differently altered in males and females under DOXO. To evaluate the cardiac phospholipid profile in doxorubicin-mediated cardiotoxicity, we performed lipidomic analysis of the hearts from male and female rats treated or not with doxorubicin for 7 weeks. To separate PL classes, we used a PVA-Sil column with a gradient program, used CAD for PL quantification and an ESI hybrid mass spectrometer (ESI/MS) to characterize the PL (Fig. 1a). The level of pooled main phospholipids (CL/phosphatidylinositol (PI)/phosphatidylethanolamine (PE)/phosphatidylserine (PS)/phosphatidylcholine (PC)) was similar in basal condition for both sexes; however, after doxorubicin treatment, cardiac phospholipid content was decreased only for the males (Fig. 1b). Principal component analysis of variables from LC-MS/MS analysis showed that non-treated animals clearly separate from doxorubicin-treated animals (21.2 % of variance explained on principal component PC1), and males trend to separate from females (10.9 % of variance explained on PC3) (Fig. 1c). These analyses are in accordance with our previous results demonstrating sex difference in DOXO cardiotoxicity [5]. The MS data were then subjected to orthogonal partial least squares discriminant analysis (OPLS-DA) in order to detect the variables which characterise the most important changes between male and female in non-treated condition or after doxorubicin treatment and the effect of treatment in each sex (Additional file 1: Table S1). We found mainly discriminating PE and CL species and less species of PC, phosphatidylglycerol (PG), and monolysocardiolipin (MLCL). Of note was the number of PL species with longer chains (≥20 carbons) showing sex differences. These results underline variation of cardiac phospholipid composition after doxorubicin treatment and differently according to sex.

**Fig. 2:**
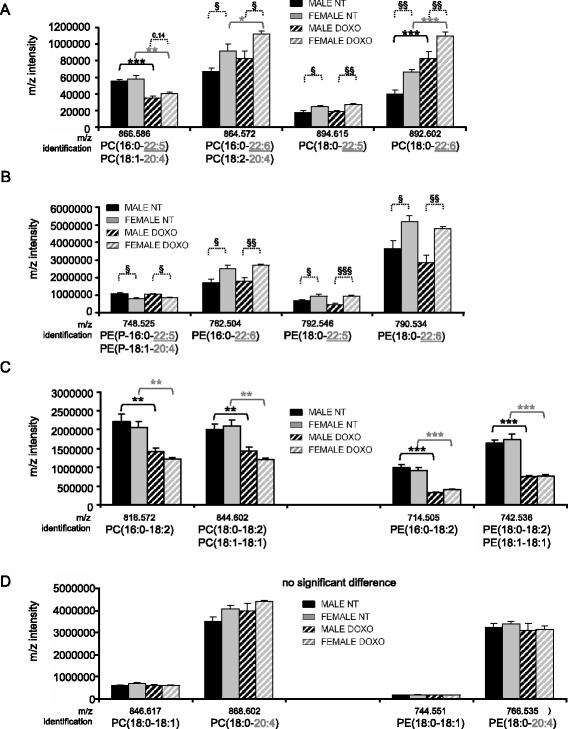
Doxorubicin and sex effect on cardiac PC and PE profile. **a** Analysis of *m*/*z* intensity of PC species by LC-ESI/MS (mean ± SEM of four rats per group, duplicated samples) with sex difference after doxorubicin treatment. **b** Analysis of *m*/*z* intensity of PE species by LC-ESI/MS with sex difference after doxorubicin treatment. **c** Doxorubicin effect (without sex difference) of *m*/*z* intensity of PC and PE. **d** No significant difference between groups of *m*/*z* intensity of PC and PE

As PE and PC are the most abundant cardiac phospholipids (Fig. 1a), we then analyzed in more detail their fatty acyl chain composition. As shown in Fig. 2a, important sex differences are present at baseline and after DOXO for PC containing docosapentaenoic (22:5) and docosahexaenoic (22:6) acyl chains. Interestingly, the same profile was observed for the PE species with similar acyl chains (Fig. 2b). In contrast, PE and PC consisting of linoleoyl acyl chain (18:2) combined with saturated palmitic or stearic acyl chain did not present sex difference but were strongly reduced by DOXO treatment in both sexes (Fig. 2c). Finally, two other PE and PC species presented no change among sex and treatment (Fig. 2d). These results underline a great variability of PE and PC species and show that the level of the different species may vary according to doxorubicin and/or sex.

### Sex-specific cardiolipin remodelling after doxorubicin treatment

**Fig. 3:**
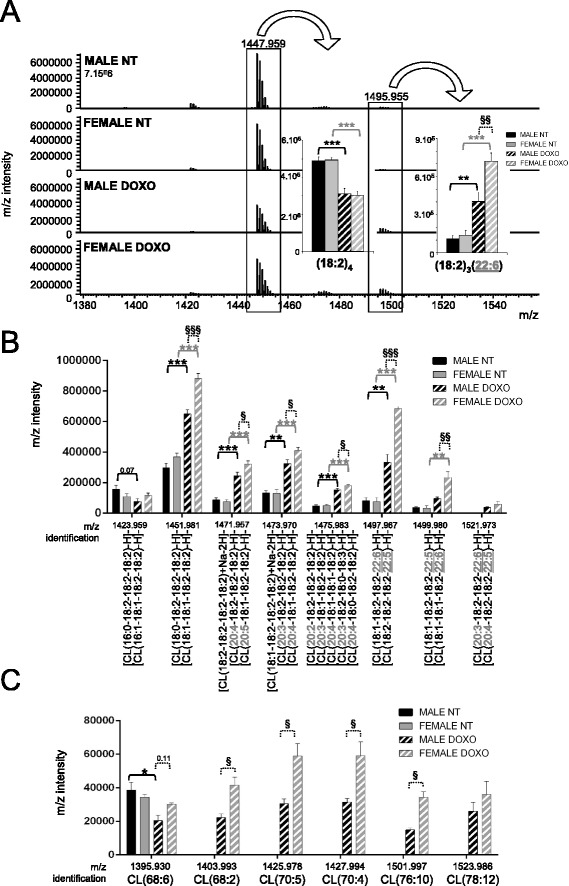
Sex-specific cardiolipin remodelling after doxorubicin treatment. **a** Cardiolipin spectra obtained by LC-ESI/MS from male and female rats treated for 7 weeks with doxorubicin (DOXO) or saline (NT). Insert panels: mean intensity of CL(18:2)_4_ and CL(18:2)_3_(22:6). **b** Identified cardiolipin *m*/*z* intensity (mean ± SEM of four rats per group, duplicated samples). **c** Non-identified cardiolipin intensity mainly present in doxorubicin-treated rats

As we previously showed, a decrease in CL content particularly in DOXO-treated males [5], analysis of cardiolipin species was necessary to dissect this deficit. Because of the four acyl chains, a great variety of CL was identified (Additional file 1: Table S2). A marked reduction of the most abundant CL: the tetra-linoleoyl (18:2)_4_ (L_4_CL) was observed after DOXO treatment but with no differences between sex (Fig. 3a). In contrast, almost all the other CL species were more abundant after DOXO treatment, and even more for the females (Fig. 3b), which may compensate for the tetra-linoleoyl CL loss. Doxorubicin induced a profound remodelling of CL species with longer acyl chains (≥20 carbons). Some cardiolipin species were detected only after doxorubicin (Fig. 3c). Moreover, sex difference occurred only after doxorubicin treatment, as no basal male-female difference was observed (Fig. 3a–c). These results showed the complexity of CL profile in the heart. As summarized in the Table 2, the cardiotoxic treatment induced a severe remodelling in the CL family and the formation of new CL species and differentially between sex, as shown by the interaction between sex and doxorubicin treatment specific for cardiolipin.

**Table 2:**
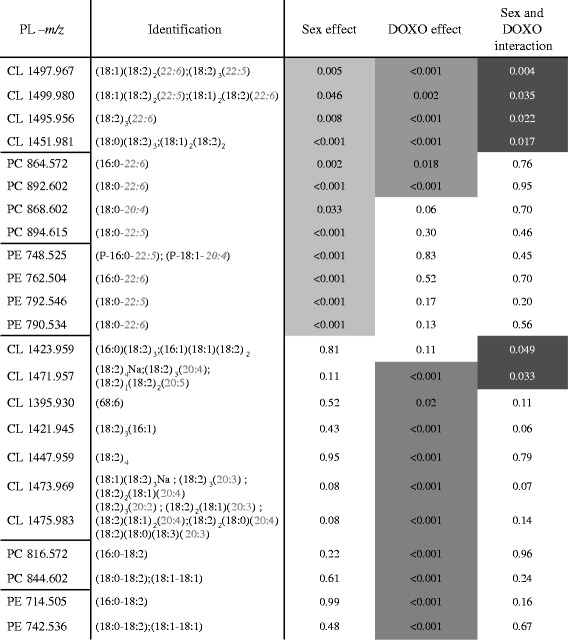
Statistical analysis of sex, doxorubicin treatment, and interaction between sex and treatment for the three main phospholipids

### Low level of oxidized cardiolipin after 7 weeks of doxorubicin treatment in males

**Fig. 4:**
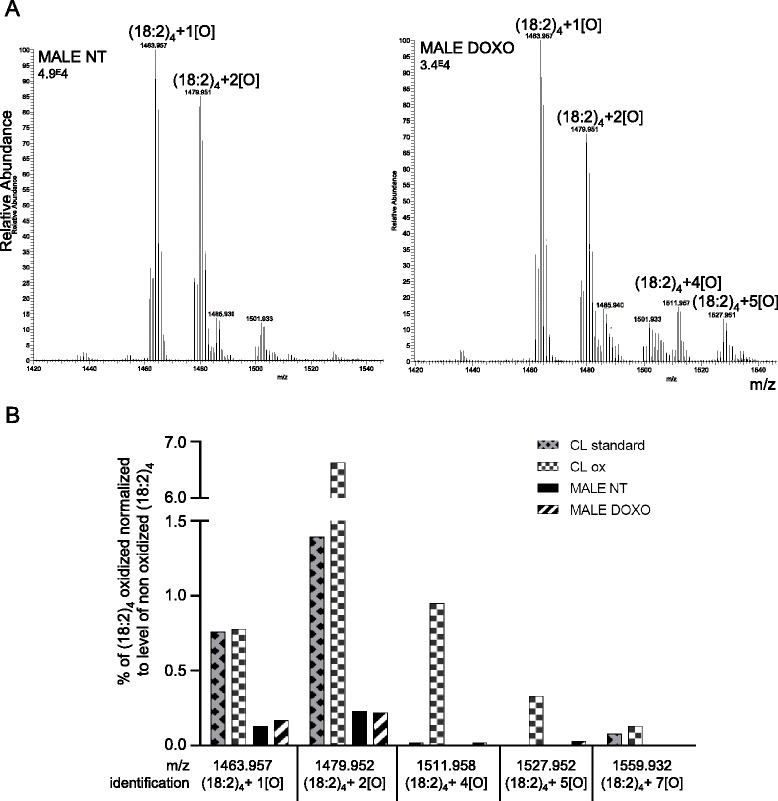
Low level of oxidized cardiolipin after 7 weeks of doxorubicin treatment in males and females. **a** Second dimension chromatographic separation of cardiolipin collected on the first dimension: full scan analysis by LC-ESI/MS of one non-treated male and one doxorubicin-treated male. **b** Relative representation of oxidized cardiolipin

Because cardiolipin oxidation has been observed in different pathologies and doxorubicin is a well-known stress oxidant inducer, we analyzed CL oxidation. Based on a previous study [31], we performed a two-dimensional HPLC coupled to MS to separate oxidized CL from non-oxidized counterparts. As a positive control, we oxidized standard CLs (CLox) with oxygenated buffer containing ferrous sulfate, ADP, and ascorbic acid. We verified that the CLox were part of the cardiolipin peak in the first dimension (Additional file 1: Figure S1A). In the second dimension, CLox were resolved into multiple peaks (Additional file 1: Figure S1B and S2A, B). The tetra-linoleoyl (*m*/*z* 1447.9) CL was oxidized in various species (i.e., *m*/*z* 1463.9, 1479.9, 1495.9, and 1511.9 corresponding to the addition of 1 to 4 oxygens). In the heart sample of non-treated or doxorubicin-treated males, we could detect CLox; however, the level of these species were very low (<0.5 %; Fig. 4a, b). Even if the percentage of CL + 4[O] and CL + 5[O] appeared slightly more important in the DOXO-treated males than in the non-treated ones, the global level of oxidation seemed rather minimal. For this reason, we did not analyze the CLox in the female animals. Overall, these results suggest that oxidative damage to cardiac cardiolipin is not largely found after 7 weeks of doxorubicin treatment. These data are in accordance with our previous results showing that global protein carbonylation was comparable in the heart of males and females treated or not with doxorubicin [5].

### Cardiolipin biosynthesis is altered in males and females treated with doxorubicin

**Fig. 5:**
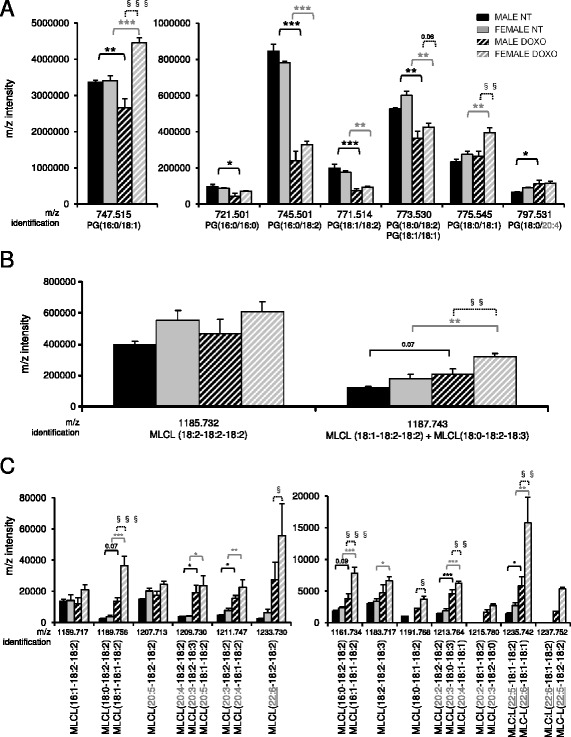
Doxorubicin and sex effects on cardiac PG and MLCL profiles. **a** Analysis of *m*/*z* intensity of PG species by LC-ESI/MS (mean ± SEM of four rats per group, duplicated samples). **b** Analysis of *m*/*z* intensity of MLCL species by LC-ESI/MS

CL biosynthesis is a multi-step process and the nascent CL from phosphatidyl glycerol (PG) is remodeled by different enzymes to form a “mature” CL (Additional file 1: Figure S3). Another important remodelling pathway includes the conversion of MLCL to CL. Moreover, a link has been made between the content of PL(18:2) and the poly-unsaturated FA metabolism [32]. Thus, we analyzed the PG and MLCL cardiac content. We identified only eight PG species (Additional file 1: Table S2 and Fig. 5a). In contrast to PE and PC, we did not detect PG containing long acyl chains. However, similar to PE and PC, PG consisting of linoleoyl acyl chains (18:2) combined with saturated palmitic or stearic acyl chains did not present sex difference but were strongly reduced by doxorubicin treatment in both sexes (Fig. 5a). To note, PG (16:0-18:1) was specifically upregulated in doxorubicin-treated females. Concerning MLCL, we identified 26 different members (Additional file 1: Table S2) with the most abundant form: the tri -linoleoyl MLCL (18:2)_3_ (Fig. 5b). Surprisingly, the level of this MLCL did not vary between groups. However, similar to CL, doxorubicin induced a sex-specific remodelling of MLCL species with longer acyl chains (Fig. 5c).

**Fig. 6:**
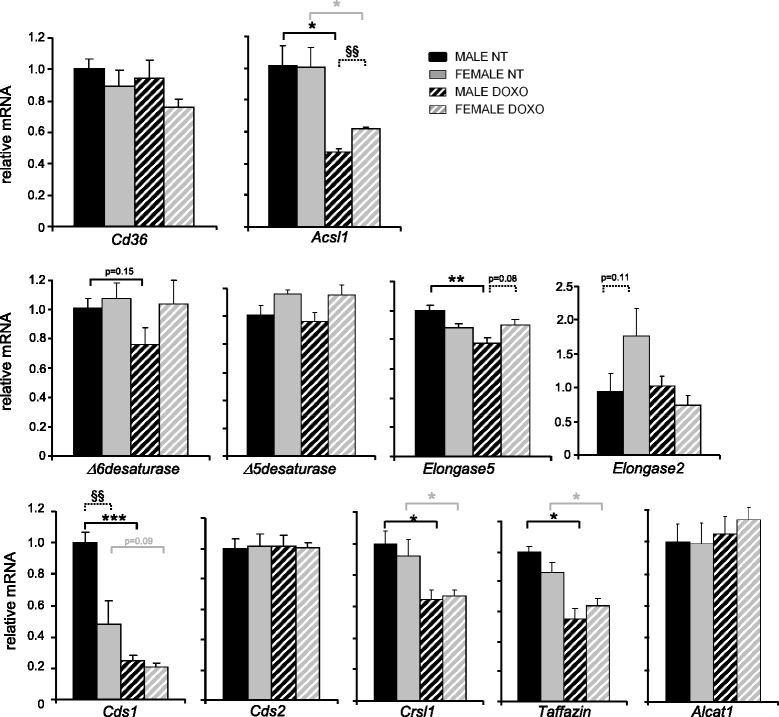
Fatty acid synthesis and cardiolipin homeostasis pathway analysis in males and females treated with doxorubicin. Gene expression analysis in ventricle of 7 weeks doxorubicin-treated rats: *Cd36*, *Acsl1*, *Cds1*, *Cds2*, *Crsl1*, *Tafazzin*, *Alcat1*, *Δ6-desaturase*, *Δ5-desaturase*, and *Elongases 2* and *5* (mean ± SEM of four rats per group)

Finally, the expression of key genes involved in phospholipid, FA metabolism, and CL remodelling was assessed (Fig. 6). No significant change was observed for the plasma membrane fatty acid transport protein *Cd36*, CDP-diacylglycerol synthase2 (*Cds2),* Acyl-CoA:lysocardiolipin acyltransferase-1 (*Alcat1*), and *Δ5-desaturase* expression among the four groups. A lower expression of *Cds1* was observed in non-treated females compared to males. Doxorubicin induced a decrease in expression of acyl-CoA synthetase long-chain family member 1 *Acsl1*, *Cds1*, cardiolipin synthase1 (*Crls1*), *Tafazzin*, *Δ6-desaturase*, and *Elongase5* for males. Regarding treated females, decreased expression was similar as males for *Cds1* and *Crl1* and less important for the other genes, except for *Δ6-desaturase* and *Elongase5* as no reduction was observed. Taken together, these results show a sex-specific remodelling of MLCL species with longer acyl chains in females and a more reduced expression of enzymes involved in fatty acid synthesis pathway in doxorubicin-treated males than females (Additional file 1: Figure S3).

## Discussion

In support of our previous work showing male-specific doxorubicin cardiotoxicity [5], this study describes for the first time the cardiac phospholipidome after doxorubicin treatment in both sexes. The role of phospholipids in the heart and the sex impact has been poorly explored, despite essential structure and signal transduction functions. After chronic doxorubicin treatment in male and female adult rats, we characterized doxorubicin and sex-specific phospholipid remodelling as best discriminating component by PCA.

From the 1980s, alterations of heart phospholipid content have been described in many cardiovascular diseases. Decrease in linoleic acid and increases in arachidonic and docosahexaenoic acid have been observed in myocardial phospholipids of rats and cats after TAC [33] and chronic coronary artery ligation [34]. A link has been made between alteration in PL and the severity of heart failure. PE, PC, PI, and PS, the main classes of PL, are present in different membranes of cardiomyocytes (sarcolemma, RS, mitochondria). These phospholipids are involved in various signal transduction pathways; they can be split by phospholipases into inositol triphosphate and diacylglycerol that function as second messengers. As we used heart tissue, we cannot discriminate the sarcolemma PL from the intracellular membrane organelles PL. Additional investigations are needed to determine the exact composition of the different membrane organelles. Thus, we will focus the discussion on CL because there are specific to the inner mitochondrial membrane and appears critical for mitochondrial function and cardiac pathology [9].

Several studies have highlighted the essential role of the mitochondria in heart failure and in anthracycline cardiotoxicity [5, 35–37]. The energetic depletion linked to mitochondrial defect is often related to a reduction in mitochondrial mass. However, when maximal oxygen consumption decreased and activities of key enzymes of energy metabolism (e.g., CS, CK, and COX) remained stable with no change in protein level of the peroxisome proliferator-activated receptor gamma co-activator 1 (PGC-1), the orchestrator for mitochondrial biogenesis, mitochondrial energetic deficiency has been suggested to be linked to a disruption of local environment [5, 38]. One key parameter for optimal and functional mitochondria is cardiolipin. Not surprisingly, CL alterations such as content, distribution, oxidation, and acyl chain composition have been reported in many diseases including Barth syndrome, Parkinson disease, and heart failure [14]. As previously described [13], the main molecular species of cardiolipin is the L_4_CL (18:2)_4_, representing around 55 % of total CL in male and female rat hearts. This level was reduced to 35 % in both sexes after doxorubicin treatment. Decrease of linoleic acid in cardiolipin has also been observed in ischemic, dilated, diabetic cardiomyopathies, and in senescent heart [20, 39, 40]. Moreover, most of the time, increase in 22:6 docosahexaenoic acid was noticed [20, 39, 40]. Hearts from doxorubicin-treated males presented an increase in CL containing 22:6 acyl chain, and this was highly exacerbated for the females. Moreover, some CL species appeared in DOXO-treated animals that were not present in controls also with a significantly higher content in females, indicating the occurrence of an alternate form of CL remodelling after DOXO treatment. Such a cardiac long acyl chain PL remodelling has been reported in heart failure and aging [32, 41]. One important point is that all these studies showing acyl chain CL remodelling were obtained in male animals/patients, and no comparison was made with females. This remodelling in males might be a compensatory mechanism but still insufficient to correct CL alterations. Because the total CL level was not reduced in DOXO-treated females and because mitochondrial function was not altered [5], it can be inferred that the significant increase in longer acyl chain (20:2, 20:3, 20:4, 22:5, and 22:6) could have compensated for the loss of (18:2)_4_ CL thus preserving the local environment for normal mitochondrial function. Indeed, calcium retention, cytochrome c sequestration, membrane fusion and fission, respiratory chain, and apoptosis are all dependent on CL content [9–12].

Because one of the controversial molecular mechanisms of anthracycline-induced cardiotoxicity is oxidative stress [42–44], we used a recently developed two-dimensional LC-MS/MS [28, 29] to analyze oxidized CL. Various conditions can cause cardiolipin oxidation, among them cytochrome c/H_2_O_2_ and oxygenated Fe2^+^ buffer (hydroxyl radical generated by the Fenton and the Haber-Weiss) reactions [28, 31, 45]. As doxorubicin leads to accumulation of iron in the mitochondria [46], we used ferrous sulfate buffer for CLox-positive control. We did not observe a high level of CLox in doxorubicin-treated males. Indeed, around 0.05 % of (18:2)_4_ containing 4 to 7 oxygens was detected. This result is in accordance with our previous data showing no obvious global protein oxidation [5]. Obviously, oxidative stress is species-, time-, and drug concentration-dependent, in other words model-dependent [42–44, 46–48]. It seems that while high level of CLox has been observed in acute stress, level of CLox could be moderate in chronic stress. After traumatic brain injury in postnatal day 17 rats, the content of oxidized CL species increased 20-fold at the expense of non-oxidized species [49]. Chronic western diet of LDLR−/− (low density lipoprotein receptor knockout) mice during 16 weeks induced a 0.1 % significant increase of oxidized cardiolipin in the liver mitochondria [50]. Therefore, we can hypothesize that either chronic doxorubicin treatment with moderate doses induces only low levels of CLox or that CLox may be produced early after injection but rapidly discarded to avoid mitochondrial dysfunction.

In DOXO-treated males but not females, we observed an early decrease in PGC-1β [5]. Recently, PGC-1α/β, key transcriptional regulators of mitochondrial metabolism, has been implicated in the regulation of cardiac phospholipid biosynthesis [51]. Despite grossly normal cardiac function of PGC-1α/β heart−/−, mitochondrial ultrastructure abnormalities were observed [51]. PGC-1α/β hearts−/− were characterized by normal level of MLCL but reduced levels of most PE, PC, and CL and a decreased level of CDP-diacylglycerol synthase *Cds-1* showing that *Cds-1* was a direct target of PGC-1. We observed that *Cds-1* was highly downregulated after doxorubicin in both sexes and even in basal level for females. In PGC-1α/β heart −/−, a reduction in PL-containing 22:5 and 22:6 acyl chains was observed [51] whereas in our model an increase was noticed. Thus, PL remodelling in DOXO cardiotoxicity can hardly be explained by PGC-1 downregulation.

It has been proposed that remodelling of CL can be linked to poly-unsaturated fatty acid metabolism [32]. An interesting feature of female cardiac PLs is the presence of species with long acyl chains not only in CL but in other PL classes. Elevated long-chain omega 3 fatty acids in females under the same diet than males have been reported in numerous studies [52]. This sex difference has been linked in part to estrogens, FA remodelling enzymes, and peroxisome proliferator-activated receptor alpha (PPARα) activity. In the liver, but not the heart, gene expression of *Δ6-* and *Δ5-desaturases* and *Elongases*, enzymes involved in the production of long-chain poly-unsaturated FAs from 18:2, is greater in females [53]. In our study, supply of essential fatty acid (α-linolenic acid 18:3 and linoleic acid 18:2) from diet was the same for males and females treated or not with doxorubicin. However, in males, the specific decrease of *Elongase5* and the tendency to decrease for *Δ6-desaturase* were more suggestive of impaired rather than improved production of long-chain fatty acids from α-linolenic acid 18:3 and linoleic acid 18:2. Expression of these enzymes has been shown to be under the control of an estrogen-mediated PPARα activation. Interestingly, we have previously observed that PPARα was less downregulated in doxorubicin-treated females [5]. Regarding FA pathway, the gene expression of *Cd36,* a regulator of the first step, i.e., uptake of FA across the plasma membrane, remained stable after DOXO in both sexes. However, *Acsl1*, the major acyl-CoA synthethase isoform in the heart that converts long-chain fatty acid into acyl-CoA thioesters plus AMP was also less downregulated in females. Interestingly, ACSL1 in the liver was described to play an important role in directing FA into pathways of phospholipid synthesis and away from cholesterolesterification and β-oxidation [54]. After doxorubicin treatment, we have shown that multiple steps of β-oxidation (MCAD and CPT1) were downregulated similarly in both sexes [5]. It appeared that FA pathway of PL synthesis rather than β-oxidation seems more preserved in females that have a better cardiac function (Additional file 1: Figure S3). In line with all these results, we have previously observed that females treated with doxorubicin have intensified liver hepatomegaly and elevated circulating level of triglycerides compared to males [5]. Similar to our results, Julicher et al. also observed a more important hepatomegaly after doxorubicin treatment in females [55]. We can hypothesize that a protective mechanism linked to lipid metabolism is activated only in females.

Finally, sex difference in cardiotoxicity of doxorubicin has not been extensively studied. However, some clinical and experimental studies have observed that females develop less unwanted cardiac side effects after doxorubicin treatment than males [5, 55–59]. The mechanism of female cardioprotection is far from being completely understood. Among the proposed pathways involved were higher oxidative defenses capacity in females [57], increased level of cardiac mast cells activity in males [59], and better mitochondrial metabolism and bioenergetic protection in females [5]. Interestingly, interactions between doxorubicin and negatively charged phospholipid and more specifically cardiolipin have been demonstrated by several groups [2, 4]. Cardiolipins are essential for respiratory chain functioning, ATP generation, and energy transfer. The heart is a highly energy-consuming organ, and cardiac contraction is linearly related to mitochondrial respiration thus anthracycline-mediated cardiolipin alterations would be a determinant factor in mitochondrial alterations and cardiotoxicity [5, 37, 44, 60]. We have now shown that cardiolipin remodelling is probably important in the sexual dimorphism of doxorubicin-induced cardiotoxicity. Additional studies are necessary to better understand the roles of lipids in the anthracycline cardiotoxicity and sex differences.

## Conclusions

In this study, we showed that (1) a decrease of global phospholipids was only observed in doxorubicin-treated males, (2) the level of PE and PC species containing long acyl chains was more important in females than in males with or without doxorubicin, (3) doxorubicin mediated a decline of PE and PC containing one linoleoyl acyl chain in both sexes, (4) significant loss of L_4_CL was comparable in treated males and females while (18:2)_3_ MLCL remained stable, (5) the level of most long-chain CL and MLCL species was more important specifically for doxorubicin-treated females, (6) CL oxidation did not appear to be a key parameter in this chronic treatment, and (7) sex differences were found in gene expression of FA biosynthesis after doxorubicin treatment. Altogether, the cardiac phospholipidomic analysis has highlighted an interesting sex-specific PL molecular species remodelling (Table 2).

This study has unraveled a significant sexual dimorphism in the cardiac phospholipid content after a chronic cardiotoxic stress. A cause-and-effect relationship between altered phospholipid content and sex differences in doxorubicin-induced cardiotoxicity remains to be established. Further studies are needed to decode the male-specific cardiotoxicity to be able to develop new therapeutic strategies.

## Additional file

Additional file 1:
**Expanded method section. Figure S1:** Detection of oxidized cardiolipin. **Figure S2: **LC-MS/MS analysis of tetralinoleoyl cardiolipin with 1 to 4 oxygens. **Figure S3:** Speculative model of sex-specific cardiac cardiolipin remodelling after doxorubicin treatment. (PDF 171 kb)

## Abbreviations

CLs: cardiolipins
HF: heart failure
L_4_CL: tetra-linoleoyl cardiolipin (18:2)_4_
LC: liquid chromatography
MLCL: monolysocardiolipin
MS: mass spectrometry
PC: phosphatidylcholine
PE: phosphatidylethanolamine
PG: phosphatidylglycerol
PI: phosphatidylinositol
PL: phospholipid
PS: phosphatidylserine
ROS: reactive oxygen species
