# Supplementary material for: Sex-specific cardiac cardiolipin remodelling after doxorubicin treatment
Source: Biol Sex Differ. 2015 Oct 15;6:20. doi: 10.1186/s13293-015-0039-5 (PMC4608149; doi:10.1186/s13293-015-0039-5)

Additional files

Methods

Doxorubicin animal model

A chronic model of doxorubicin (1) by weekly intravenous injection of 2 mg/kg doxorubicin or saline solution during 7 weeks in 11 weeks old male and female Wistar rats (Janvier Labs) was chosen as previously described (2). Rats were housed 3 or 4 per cage in a temperature-controlled room, with a 12/12 h light/dark cycle, and were provided food and water *ad libitum*. Animal experimental procedures were approved by the Animal Ethics Committee of Paris Sud University. Investigations were done in accordance with European Community legislation relating to the care and use of animals (Directive 2010/63/EU), and the corresponding French legislation (French decree 2013-118 du 1er février 2013). At the end of the treatments, all rats were anesthetized by intraperitoneal injection of pentobarbital (200 mg/kg). The depth of anesthesia was checked by toe pinch before the start of surgery. The heart was then quickly removed for euthanasia. Part of the hearts was rapidly frozen and kept at −80°C for further investigations.

Reagents

Standards were purchased from Sigma-Aldrich with a purity of approximately 98%; cardiolipin (CL) sodium salt from bovine heart, L-α-phosphatidylglycerol (PG) form egg yolk lecithin, L-α-phosphatidylethanolamine (PE) and L-α-phosphatidylcholine (PC) from egg yolk. They were solubilized in a chloroform/methanol (2/1) mixture at a concentration of 5.0 mg/mL and then diluted in chloroform at 0.5, 0.4, 0.3, 0.2, 0.1, 0.05 and 0.025 mg/mL for the quantification. Solvents used for chromatography were N-heptane, chloroform (HPLC-grade) and methanol (LC/MS grade) from VWR International ; isopropanol and water (ULC-MS grade) from Biosolve Chemicals and acetic acid (LC-MS grade) and triethylamine (99.5%) from Sigma-Aldrich.

Real-time quantitative PCR analysis

Total ventricular RNA was extracted using Trizol reagent (Invitrogen). Two µg of total RNA was used to synthesize cDNA according to the protocol provided with the High Capacity cDNA Reverse Transcription Kit (Applied Biosystems, France). Quantification of mRNA via production of cDNA was assessed using the SYBR Green method as previously described (3)

on a CFX96 (Bio-Rad). The following primers were used: *Ywhaz* (5'AGACGGAAGGTGCTGAGAAA3' ; 5'GAAGCATTGGGGATCAAGAA3'), *PolR2a* (5'CAACATGCTGACAGATATGACC3' ; 5'TGATGATCTTCTTCTTGTTGTCTG3'), *Cd36* (5'CCACTGTGTACAGACAGTTTTGG3' ; 5'TGTTTGCCTTCTCATCACCA3'), *Acsl1* (5'ATGAAGGCTACGGACAGACC3' ; 5'CTTTCACACACACCTCACCC3'), *Crsl1* (5'TGACCTATGCAGATCTTATTCCA3' ; 5'CCTTGCTGATGAATGTTGGTT3'), *Cds2*, *Taffazzin* and *Alcat1* (4), *Cds1* (5), *Δ6Desaturase-Δ5Desaturase-Elongase5-Elongase* 2 (6). mRNA levels for all target genes were normalized to *Ywhaz, PolR2a* and *Rplp2* levels using GeNorm software (7).

Figure legends:

Figure S1: Detection of oxidized cardiolipin

A. Oxidized cardiolipin was subjected to 1st dimension phospholipid class separation obtained by LC-ESI/MS. Total ion chromatogram and cardiolipin spectra are represented. B. Oxidized cardiolipin was collected from 11 to 12 min during the 1st dimension, dried over nitrogen, and dissolved in 2D phase mobile before being subjected to 2nd dimension.

Figure S2: LC-MS/MS analysis of tetralinoleoyl cardiolipin with 1 to 4 oxygens.

A. Specific full scan m/z screaning of $(18:2)_4$+n[O]. B. Specific spectra of $(18:2)_4$+n[O].

Figure S3: Speculative model of sex-specific cardiac cardiolipin remodelling after doxorubicin treatment.

Doxorubicin induced a decrease in expression of acyl-CoA synthetase long-chain family member 1 *Acsl1,* only for males. ACSL1 plays an important role in directing FA into pathways of phospholipid synthesis and β-oxidation. FA pathway of PL synthesis rather than β-oxidation seems more preserved in females. Similarly, Elongase5 and Δ6-desaturase which play critical roles in regulating the length and degree of unsaturation of fatty acids, are specially altered in doxorubicin-treated males. Finally, while the most abundant CL, the tetra-linoleoyl cardiolipin was decreased for both male and female after doxorubicin, total CL is male specific lower (as shown in our previous study Moulin *et al*., 2015). Doxorubicin-treated females have compensating increased in CL containing long acyl chain.

References

1. Lightfoot JC, D'Agostino RB, Jr., Hamilton CA, Jordan J, Torti FM, Kock ND, et al. Novel approach to early detection of doxorubicin cardiotoxicity by gadolinium-enhanced cardiovascular magnetic resonance imaging in an experimental model. Circ Cardiovasc Imaging. 2010;3(5):550-8.
2. Dworatzek E, Mahmoodzadeh S, Schubert C, Westphal C, Leber J, Kusch A, et al. Sex differences in exercise-induced physiological myocardial hypertrophy are modulated by oestrogen receptor beta. Cardiovascular research. 2014;102(3):418-28.
3. Piquereau J, Novotova M, Fortin D, Garnier A, Ventura-Clapier R, Veksler V, et al. Postnatal development of mouse heart: formation of energetic microdomains. The Journal of physiology. 2010;588(Pt 13):2443-54.
4. Athea Y, Viollet B, Mateo P, Rousseau D, Novotova M, Garnier A, et al. AMP-activated protein kinase alpha2 deficiency affects cardiac cardiolipin homeostasis and mitochondrial function. Diabetes. 2007;56(3):786-94.
5. Lai L, Wang M, Martin OJ, Leone TC, Vega RB, Han X, et al. A role for peroxisome proliferator-activated receptor gamma coactivator 1 (PGC-1) in the regulation of cardiac mitochondrial phospholipid biosynthesis. The Journal of biological chemistry. 2014;289(4):2250-9.
6. Le CH, Mulligan CM, Routh MA, Bouma GJ, Frye MA, Jeckel KM, et al. Delta-6-desaturase links polyunsaturated fatty acid metabolism with phospholipid remodeling and disease progression in heart failure. Circulation. 2014;7(1):172-83.
7. Vandesompele J, De Preter K, Pattyn F, Poppe B, Van Roy N, De Paepe A, et al. Accurate normalization of real-time quantitative RT-PCR data by geometric averaging of multiple internal control genes. Genome biology. 2002;3(7):RESEARCH0034.

Table S1

Identification of potential phospholipids for sex and/or doxorubicin difference by OPLS-DA analysis

	m-z	retention time	correlation	IDENTIFICATION
NT M vs F	892,603	19,649	0,89	PC(18:0-22:6)
MALE (DOXO vs NT)	1454,000	11,38	0,95	CL(18:0-18:1-18:2-18:2)
	1404,000	11,48	0,95	CL(68:2)
	1548,080	14,29	0,96	PE(P-18:0-22:6)
	1506,050	14,60	-0,92	PE(18:0-18:2) PE(18:1-18:1)
	742,537	14,66	-0,96	PE(18:0-18:2) PE(18:1-18:1)
	714,506	14,77	-0,95	PE(16:0-18:2)
	716,521	14,78	-0,94	PE(16:0-18:1)
	1432,030	14,79	-0,95	PE(16:0-18:1)
	1494,020	10,30	-0,93	PG(16:0-18:1)
	745,501	10,30	-0,96	PG(16:0-18:2)
	866,587	19,73	-0,92	PC(18:1-20:4) PC(16:0-22:5)
FEMALE (DOXO vs NT)	1497,970	11,29	0,96	CL(18:1-18:2-18:2-22:6) CL(18:2-18:2-18:2-22:5)
	1495,960	11,29	0,97	CL(18:2-18:2-18:2-22:6)
	1471,960	11,31	0,97	CL$(18:2)_4$+Na CL(20:4-18:2-18:2-18:2) CL(20:5-18:1-18:2-18:2)
	1454,000	11,38	0,95	CL(18:0-18:1-18:2-18:2)
	1451,980	11,38	0,97	CL(18:0-18:2-18:2-18:2) CL(18:1-18:1-18:2-18:2)
	774,541	14,31	0,95	PE(P-18:0-22:6)
	746,511	14,46	0,94	PE(P-16:0-22:6)
	1506,050	14,60	-0,94	PE(18:0-18:2) PE(18:1-18:1)
	740,521	14,64	-0,97	PE(18:1-18:2)
	1454,020	14,73	-0,93	PE(16:0-18:1)
	714,506	14,77	-0,94	PE(16:0-18:2)
	771,515	10,23	-0,95	PG(18:1-18:2)
	745,501	10,30	-0,96	PG(16:0-18:2)
	866,587	19,73	-0,93	PC(18:1-20:4) PC(16:0-22:5)
	818,570	19,63	0,94	PC(16:0-18:1) PC(18:0-22:5)
	1658,170	19,78	0,94	PC(16:0-18:1) PC(18:0-22:5)
DOXO (MALE vs FEMALE)	1451,980	11,38	0,90	CL(18:0-18:2-18:2-18:2) CL(18:1-18:1-18:2-18:2)
	1215,780	13,30	0,89	MLCL(20:2-18:1-18:2) MLCL(20:3-18:2-18:0)
	816,551	14,23	0,89	PE(42:7)
	1532,060	14,49	0,89	PE(18:0-20:4)
	1524,080	14,33	-0,87	PE(18:0-18:1)
	1498,060	14,43	-0,87	PE(16:0-20:4) PE(18:2-18:2)
	1496,040	14,44	-0,88	PE(P-16:0-22:5) PE(P-18:1-20:4)
	1522,060	14,34	-0,86	PE(P-16:0-22:4) PE(P-18:0-20:4)
	1556,060	14,40	0,92	PE(P-40:4)
	1480,030	14,70	0,93	PE(P-16:0-20:4)

Table S2

	Total species	Species identified
**PG**	8	8
**CL**	36	30
**MLCL**	26	26
**PE**	21 PE<br>9 PE plasmalogene	17 PE<br>9 P-PE
**PC**	22 PC<br>4 PC plasmalogene	20 PC<br>0 P-PC

Supp Figure 1

A

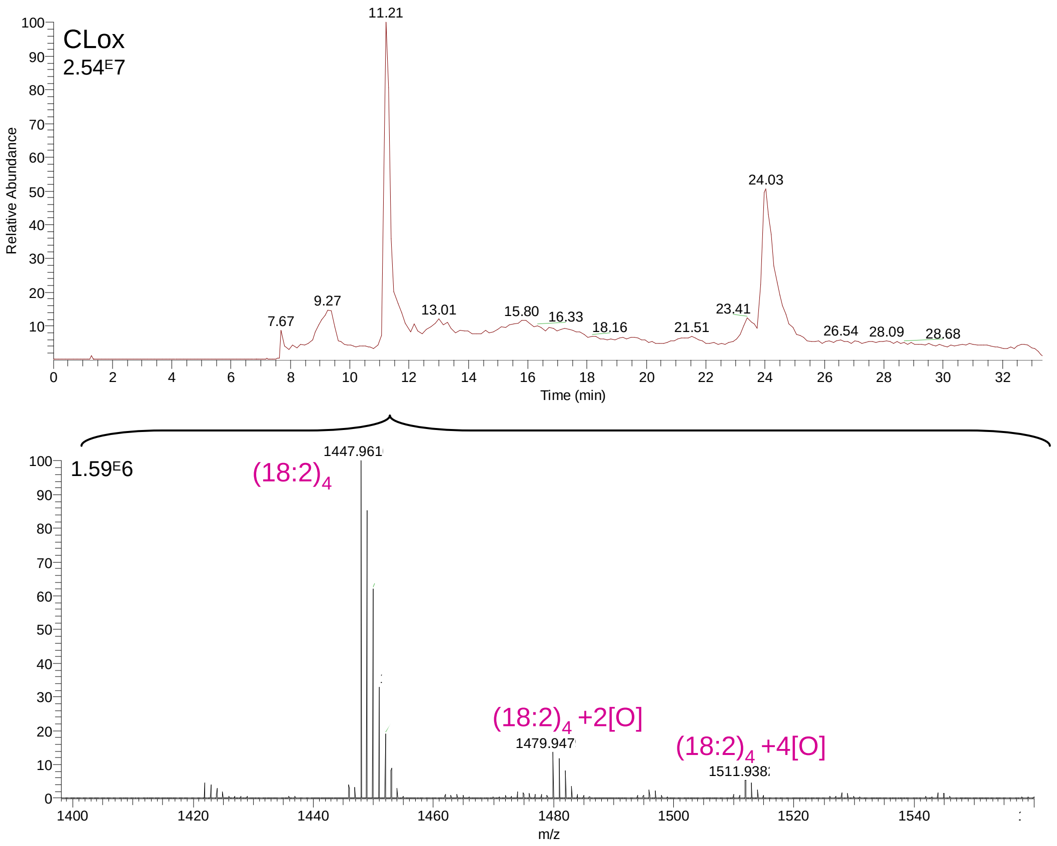

B

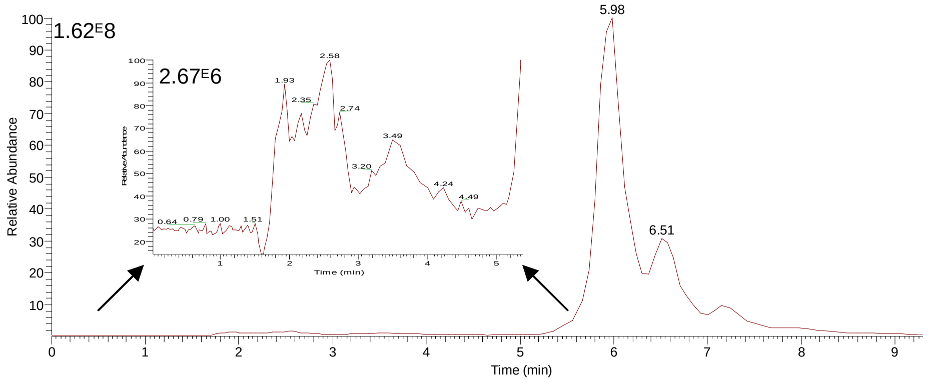

Supp Figure 2

A

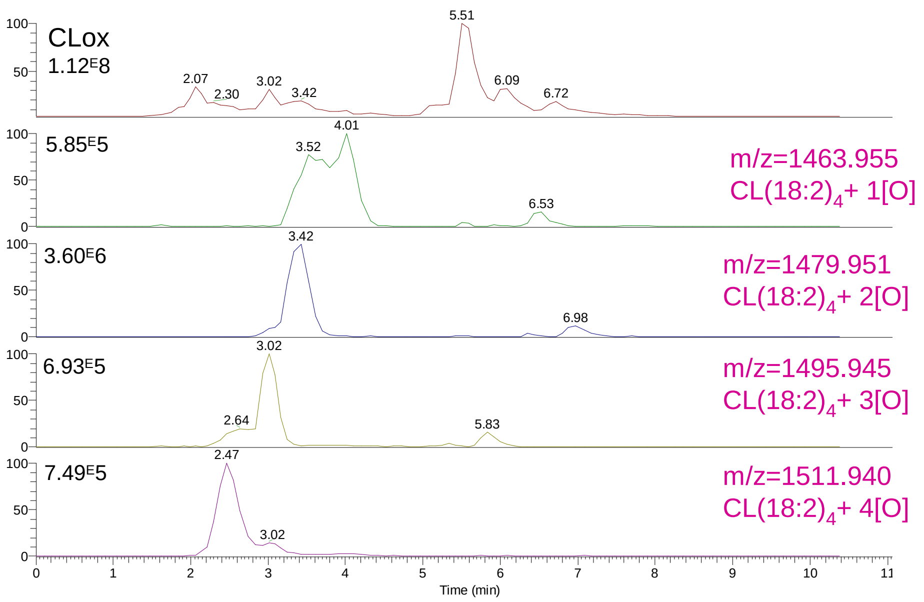

B

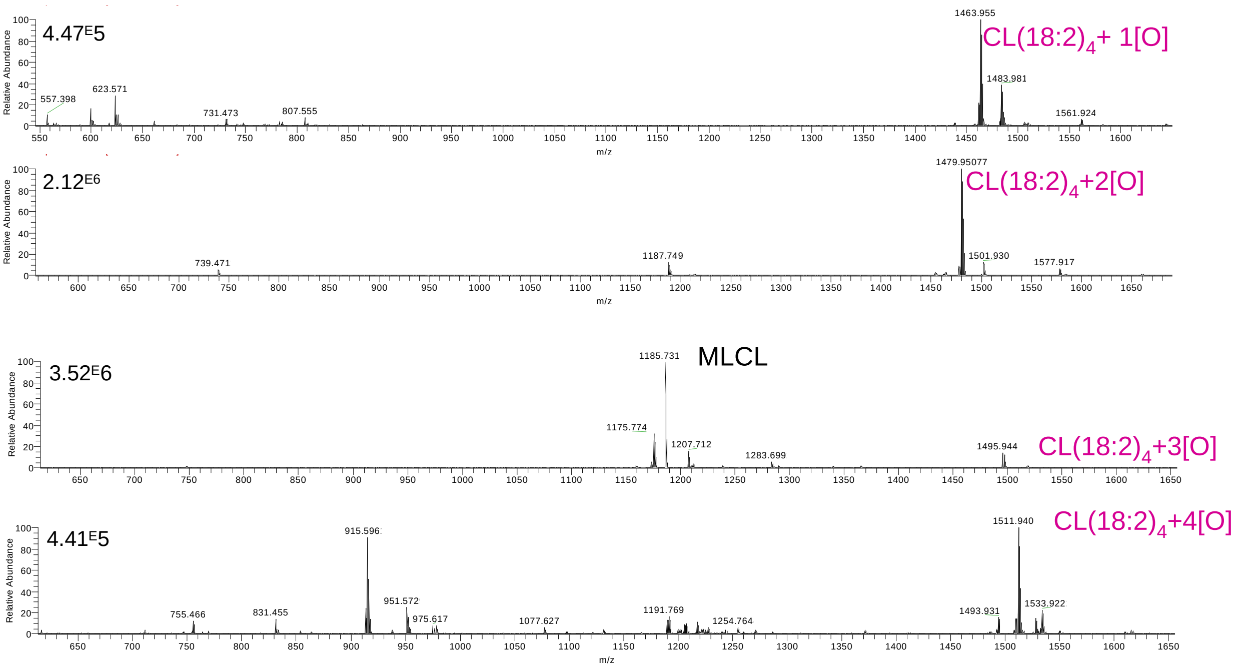

Supp Figure 3

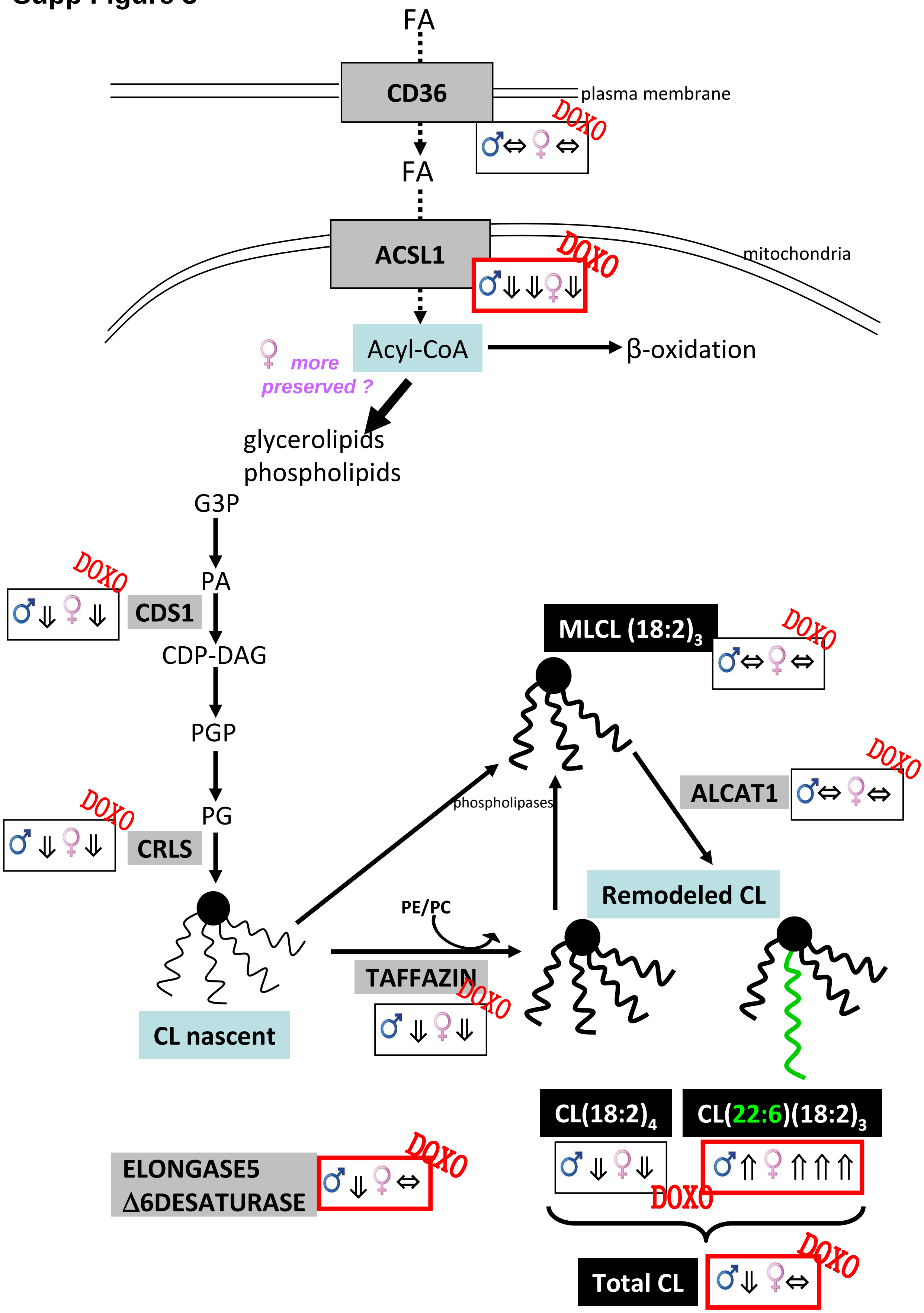

Supplement: Additional file 1: — Expanded method section. Figure S1: Detection of oxidized cardiolipin. Figure S2: LC-MS/MS analysis of tetralinoleoyl cardiolipin with 1 to 4 oxygens. Figure S3: Speculative model of sex-specific cardiac cardiolipin remodelling after doxorubicin treatment. (PDF 171 kb) [file 13293_2015_39_MOESM1_ESM.pdf]
